# Environmental Regulation of Gut Microbial Networks Links to Growth Variation in *Schizopygopsis younghusbandi* Across Contrasting Aquaculture Systems

**DOI:** 10.3390/microorganisms14040925

**Published:** 2026-04-20

**Authors:** Wanliang Wang, Zhuangzhuang Wang, Peng Zhang, Jifeng Zhang

**Affiliations:** 1 College of Ecological Environment, Xizang University, Lasa 850000, China; qlxlsylzfyzx@163.com (W.W.); pengzhang09@126.com (P.Z.); 2 Institute of Aquatic Sciences, Academy of Agriculture and Animal Husbandry, Xizang Autonomous Region, Lasa 850000, China; zwangzhuang@163.com

**Keywords:** *Schizopygopsis younghusbandi*, aquaculture modes, growth performance, gut microbiota, co-occurrence network

## Abstract

*Schizopygopsis younghusbandi* is an endemic and economically important fish in the Qinghai-Xizang Plateau, but its aquaculture is limited by harsh environmental conditions and incomplete understanding of host–microbiome–environment interactions. This study applied metagenomic sequencing to examine how different culture environments affect growth, water microbial communities, and gut microbiome network stability. Three-year-old juveniles (initial body weight 50.57 ± 1.88 g) were reared for 90 days in five systems: conventional pond (P), wetland (WL), concrete tank (G), river (R), and recirculating aquaculture system (RC). No significant differences in initial body weight or length were observed among groups (*p* > 0.05). Fish in the RC system achieved the highest final body weight, weight gain rate, and specific growth rate (*p* < 0.05), while survival rates were highest in the river and RC groups and lowest in ponds (*p* < 0.05). Microbial diversity and community composition differed significantly among culture modes, with bacterial and protozoan communities showing the strongest environmental responsiveness. Co-occurrence network analyses revealed that RC and G systems exhibited higher network complexity, density, and proportion of positive correlations, reflecting enhanced microbial interaction and ecological stability, whereas the WL system showed reduced network connectivity. Correlation analysis indicated that bacterial abundance was positively associated with total nitrogen, total phosphorus, and dissolved oxygen (*p* < 0.05), highlighting environmental regulation of microbial assemblages. Overall, the aquaculture environment shapes gut microbial networks, which closely relate to growth performance. Recirculating aquaculture systems can mitigate growth limitations in plateau fish by stabilizing the environment and reinforcing gut microbial communities, providing a sustainable strategy for high-altitude aquaculture development.

## 1. Introduction

China is a major aquaculture-producing country, and farmed aquatic products have substantially enriched sources of high-quality protein for human consumption, playing a significant role in ensuring national food security [1,2]. Aquaculture development in Xizang started relatively late, and plateau fish species generally exhibit slow growth, delayed sexual maturity, and long culture cycles [3], resulting in a pronounced economic gap in the fishery sector compared with other regions of China. Xizang possesses unique fishery germplasm resources, which are mainly classified into three major groups: Sisoridae, Cobitidae, and Schizothoracinae, accounting for 92.07% of indigenous fish species on the Qinghai-Xizang Plateau [4]. At present, artificial breeding technologies have been successfully developed for more than ten indigenous fish species, including *Glyptosternum maculatum*, *Schizothorax waltoni*, *Schizothorax macropogon*, and *Oxygymnocypris stewarti*. Among them, *Schizopygopsis younghusbandi* is currently the only schizothoracine species that has achieved fully artificial reproduction. It has a wide distribution and forms dominant populations in the main streams and tributaries of the middle reaches of the Yarlung Zangbo River, playing an important role in maintaining the ecological security of plateau aquatic ecosystems.

With the improvement of living standards and the vigorous development of aquaculture, the impact of the aquatic environment on fish growth and farming health is receiving increasing attention [5]. Traditional aquaculture models represented by pond culture and cage culture have been continuously optimized [6], while emerging models such as industrial recirculating aquaculture systems [7] and ecological aquaculture [8] have undergone rapid upgrading, laying a solid foundation for the diversified development of aquaculture models in China. Current aquaculture systems in Xizang mainly include open-flow culture, pond culture, and industrialized culture systems [9]. Cultured indigenous species include *Schizopygopsis younghusbandi*, *Schizothorax oconnori*, and *Salmo trutta fario* [10], while non-native species include economically important fish such as *Oncorhynchus mykiss*, hybrid sturgeons, and *Oreochromis mossambicus* [11]. Xizang is characterized by abundant water resources and high-quality water conditions, providing unique natural advantages for aquaculture development. In recent years, improvements in transportation infrastructure, rapid growth of tourism, and increased investment in fishery science and technology have jointly promoted the exploration of a distinctive and sustainable pathway for fisheries development in Xizang [12].

Different aquatic environments exhibit distinct characteristics for fish culture, and the selection of appropriate culture models and technologies is a key factor for achieving quality improvement, efficiency enhancement, and sustainable development [13]. Microorganisms play a crucial role in both natural ecosystems and human society, and they are essential components of the aquatic environment and fish gut ecosystems throughout the aquaculture process [14]. In the fish intestine, microbial communities exist in a complex and dynamic equilibrium, forming a symbiotic relationship with the host. This relationship promotes functional differentiation and complementarity among microorganisms in metabolic processes, thereby collectively supporting multiple essential physiological functions of the host, including nutrient absorption, immune regulation, physiological homeostasis, pathogen defense, and even neural activities [15,16,17]. Importantly, the gut microbiota plays a dual role in host metabolism, exerting both beneficial and potentially detrimental effects depending on its composition and stability. Specifically, the gut microbiota regulates nutrient absorption and metabolic processes through host–microbe interactions, for example, by participating in the digestion of complex carbohydrates and the regulation of lipid storage [18,19,20]. In aquaculture systems, the composition and function of fish gut microbiota are highly sensitive to habitat conditions. External environmental factors, such as salinity, nutrient availability, and rearing conditions, can significantly reshape microbial community structure, thereby influencing host growth performance and health status [21]. Meanwhile, the internal environment provided by the host also plays a crucial role in structuring microbial communities, which is in turn modulated by the resident microbiota [22]. However, the precise mechanisms underlying these bidirectional interactions between habitat, microbiota, and host metabolism remain poorly understood.

With the rapid advancement of sequencing technologies, metagenomic approaches for investigating entire microbial communities in environmental samples have developed rapidly [23], providing novel perspectives for understanding microbial diversity and function. In the field of aquaculture, these approaches have been widely applied in both marine and freshwater fish species, including *Takifugu obscurus* [24], *Ophiocephalus argus* [25], and *Gymnocypris przewalskii* [26]. High-throughput sequencing enables the characterization of microbial community dynamics in different culture environments and fish intestines, thereby facilitating the evaluation of fish health status under aquaculture conditions. As one of the important indigenous economic fish species in Xizang, research on *Schizopygopsis younghusbandi* has mainly focused on individual biology [27,28], behavioral biology [29], molecular biology [30], and genetic evolution [31]. However, studies on the effects of different aquatic environments on microbial communities during the healthy culture of *S. younghusbandi* have not yet been reported. Therefore, this study applied metagenomic techniques to compare the effects of different aquatic environmental conditions on the growth performance and gut microbial community structure of *S. younghusbandi*, and to further analyze the response patterns between environmental factors and host-associated microorganisms, aiming to provide scientific evidence and technical support for the healthy aquaculture of this species.

## 2. Materials and Methods

### 2.1. Experimental Design and Culture Management

The culture experiment was conducted in Chengguan District (Lhasa City) and Qushui County, Xizang. Five different aquatic culture environments were selected: conventional pond (P), wetland (WL), concrete tank (G), river (R), and recirculating aquaculture system (RC). Net cages with identical dimensions (1.5 m × 1.2 m × 1.2 m) were installed in each culture system. Experimental fish were first-generation offspring (F1), three-year-old juveniles obtained from the same batch at the Yarlung Zangbo River Fishery Resources Breeding Base. The fish had an average body weight of 50.57 ± 1.88 g and an average body length of 15.46 ± 1.06 cm, and were healthy, injury-free, and active. Each culture environment contained three independent replicates, and 100 individuals of *Schizopygopsis younghusbandi* were randomly stocked in each replicate. Fish were fed twice daily with Stiga-brand special extruded compound aquafeed at 10:30 a.m. and 4:00 p.m. After 20 min of feeding, uneaten feed was removed. The culture experiment lasted for 90 days.

### 2.2. Sample Collection and Index Determination

At the end of the culture experiment, feeding was suspended for 24 h prior to sampling. For each group, 6–10 water samples and gut microbiota samples were collected, with two samples randomly collected from each replicate. Water samples were filtered through 0.22 μm membrane filters (Millipore, MA, USA). All samples were immediately frozen in liquid nitrogen and stored at −80 °C. Water samples from the conventional pond, recirculating system, river, concrete tank, and wetland groups were labeled as PW, RCW, RW, GW, and WLW, respectively. Corresponding gut microbiota samples were labeled as PF, RCF, RF, GF, and WLF.

#### 2.2.1. Water Quality Measurements

Water temperature, dissolved oxygen (DO), and pH were measured in situ during sampling. Biochemical oxygen demand over five days (BOD_5_), total nitrogen (TN), total phosphorus (TP), ammonia nitrogen (NH_3_-N), nitrite (NO_2_^−^), total hardness, and total alkalinity were measured in the laboratory. Six sampling points were measured for each group, with two sampling points per net cage. Detailed water environmental parameters are shown in Table 1.

#### 2.2.2. Growth Performance Measurements

Fish were anesthetized using MS-222 at a concentration of 100 mg/mL. Growth indices were measured for 15 fish per group, with five individuals randomly selected from each net cage. Body weight was measured to the nearest 0.01 g, and body length was measured to the nearest 0.01 cm.

The growth indices were calculated as follows:

Weight gain rate (WGR, %) = 100 × (Wt − W_0_)/W_0_

Specific growth rate (SGR, %/d) = 100 × (lnWt − lnW_0_)/t

Condition factor (CF, g/cm^3^) = 100 × Wt/L^3^

Survival rate (SR, %) = 100 × Nt/N

where W_0_ is the initial body weight, Wt is the final body weight, t is the culture duration, L is the body length, Nt is the number of surviving fish, and N is the initial stocking number.

### 2.3. Collection and DNA Extraction of Intestinal Samples

After anesthetizing *Schizopygopsis younghusbandi* from different aquaculture systems using MS-222 (130 mg/L), fish were surface-sterilized with 70% ethanol, and intestinal contents were aseptically dissected and collected from approximately 1 cm anterior to the rectum under sterile conditions. The samples were immediately transferred into sterile, enzyme-free EP tubes (Servicebio, Hubei, China), placed on dry ice, and transported to the laboratory, where they were stored at −80 °C until further processing. Meanwhile, water samples (2 L) were collected using a vacuum pump（LabTech, Beijing, China） and filtered through 0.22 μm mixed cellulose ester filter membranes to capture microbial biomass. The filter membranes were subsequently transferred into sterile, enzyme-free EP tubes and stored at −80 °C until DNA extraction. Total genomic DNA was extracted from intestinal and water samples using the OMEGA DNA Kit (M5635-02) (Omega Bio-Tek, Norcross, GA, USA) according to the manufacturer’s instructions, and stored at −20 °C for downstream analyses. The concentration and purity of extracted DNA were assessed using a NanoDrop NC2000 spectrophotometer (Thermo Fisher Scientific, Waltham, MA, USA), while DNA integrity was evaluated by 1% agarose gel electrophoresis. Total genomic DNA of microbial communities was extracted using the YH-soil FastPure Soil DNA Isolation Kit (Magnetic Bead) according to the manufacturer’s instructions. After extraction, DNA concentration and purity were determined, and DNA integrity was assessed by 1% agarose gel electrophoresis. DNA was fragmented using a Covaris M220 system, and fragments of approximately 350 bp were selected for paired-end (PE) library construction. Metagenomic sequencing was performed on the Illumina NovaSeq™ X Plus platform (Shanghai Majorbio Bio-Pharm Technology Co., Ltd., Shanghai, China).

### 2.4. Metagenomic Bioinformatics

Raw metagenomic reads were quality-filtered using fastp [32] to remove adapter sequences at both 3′ and 5′ ends, as well as reads shorter than 50 bp or with an average base quality score below 20, retaining high-quality clean reads. To eliminate host contamination, clean reads were aligned against host reference sequences using BWA [33], and matching reads were removed. High-quality reads were de novo assembled using MEGAHIT [34], and contigs ≥ 300 bp were retained for downstream analyses. Open reading frames (ORFs) were predicted from assembled contigs using Prodigal [35] in metagenomic mode. All predicted genes were clustered using CD-HIT [36] with a sequence identity threshold of 90% and coverage of 90%, and the longest sequence in each cluster was selected to construct a non-redundant gene catalog. Clean reads from each sample were mapped to the non-redundant gene catalog using SOAPaligner [37] (identity ≥ 95%) to calculate gene abundance profiles. Representative amino acid sequences were annotated by aligning against the NCBI NR database using DIAMOND [38] (BLASTP, e-value ≤ 1 × 10^−5^), and taxonomic annotations were assigned based on the best hits.

### 2.5. Statistical Analyses

Water quality parameters and fish growth indices were analyzed using SPSS 20.0 software. Prior to one-way analysis of variance (ANOVA), the assumptions of normality and homogeneity of variances were tested using the Shapiro–Wilk test and Levene’s test, respectively. One-way analysis of variance (ANOVA) followed by Duncan’s multiple range test was used to evaluate differences among groups. Species annotation was obtained based on the corresponding taxonomic information in the NR database, and species abundance was calculated by summing the gene abundances corresponding to each taxon. Alpha diversity indices were calculated using the “vegan” package in R. One-way analysis of variance (ANOVA) was performed to assess the significance of associations between alpha diversity indices, network parameters, and environmental factors across different aquaculture systems. Principal coordinate analysis (PCoA) was conducted based on Bray–Curtis distances using the “vegan” package. Co-occurrence events were identified as statistically robust correlations (|R| > 0.6, *p* < 0.05), and the co-occurrence network was visualized in Gephi (version 0.10.1).

### 2.6. Animal Welfare Statement

All experimental procedures involving animals were approved by the Ethical Committee for Experimental Animals of the Xizang Autonomous Region Academy of Agricultural and Animal Sciences, Institute of Aquatic Sciences (Approval number: 2026001). All methods were carried out in accordance with the relevant guidelines and regulations. At the conclusion of the experiment, all fish were humanely euthanized in accordance with the AVMA Guidelines for the Euthanasia of Animals (2020) and the ARRIVE guidelines. Fish were first anesthetized in a buffered solution of tricaine methane sulfonate (MS-222; 150 mg/L) until loss of equilibrium and cessation of opercular movement were observed. After confirming deep anesthesia, euthanasia was completed using an overdose of MS-222 (300 mg/L) followed by pithing to ensure death. No fish regained consciousness during or after the procedure. All efforts were made to minimize animal suffering and stress throughout the study.

## 3. Results

### 3.1. Physicochemical Parameters of Different Culture Waters and Their Effects on the Growth of Schizopygopsis younghusbandi

As shown in Table 2, significant differences were observed among the different culture water environments in physicochemical parameters. Water temperature was highest in the RCW group and was significantly higher than in all other groups (*p* < 0.05). The biochemical oxygen demand over five days (BOD) was lowest in the GW group and was significantly lower than in the other groups (*p* < 0.05). Ammonia nitrogen was highest in the GW group, showing no significant difference compared with the RW group (*p* > 0.05), but was significantly higher than in the remaining groups (*p* < 0.05). Total phosphorus was lowest in the WLW group, while the RCW group showed the highest value, which was not significantly different from that of the RW group (*p* > 0.05) but was significantly higher than those of the other groups (*p* < 0.05). Total nitrogen and total hardness were highest in the GW group and were significantly higher than in all other groups (*p* < 0.05). Nitrite concentration was highest in the RCW group and was significantly higher than in the other groups (*p* < 0.05). Total alkalinity was highest in the GW group, showing no significant difference from the RW group (*p* > 0.05) but being significantly higher than in the remaining groups (*p* < 0.05). Dissolved oxygen was highest in the WLW group and was significantly higher than in all other groups (*p* < 0.05). The pH value was highest in the RCW group and lowest in the GW group, with significant differences observed among all groups (*p* < 0.05).

As shown in Table 3, different culture environments exerted distinct effects on the growth performance of *Schizopygopsis younghusbandi*. No significant differences were observed in initial body weight or initial body length among the five culture modes (*p* > 0.05). Final body weight was highest in the RCF group and was significantly greater than that in all other groups (*p* < 0.05). Final body length was lowest in the WLF group, showing no significant difference compared with the PF and RF groups (*p* > 0.05), but was significantly lower than in the remaining groups (*p* < 0.05). Both weight gain rate (WGR) and specific growth rate (SGR) reached their highest values in the RCF group and were significantly higher than those in all other groups (*p* < 0.05). The condition factor (CF) was lowest in the PF group and highest in the WLF group; except for the comparison between the GF and RF groups, significant differences were detected among all other groups (*p* < 0.05). Survival rates were highest in the RF and RCF groups and did not differ significantly from those of the GF group (*p* > 0.05), whereas the PF group exhibited the lowest survival rate, which was significantly lower than that of all other groups except the WLF group (*p* < 0.05).

### 3.2. Changes in Microbial Diversity in Different Culture Waters and in the Gut of Schizopygopsis younghusbandi

**Figure 1:**
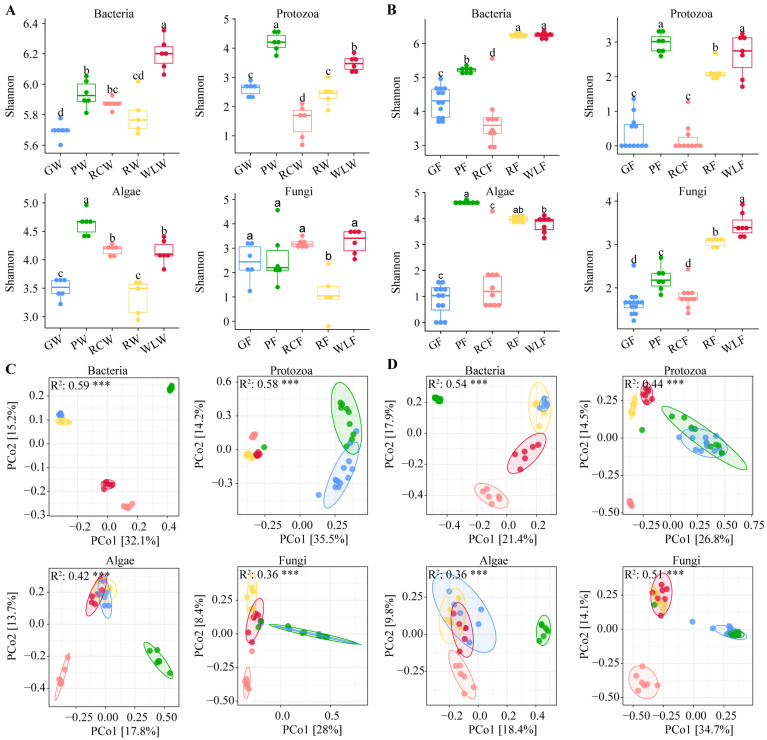
Diversity analysis of microbial communities in aquaculture water environments and the gut of *Schizopygopsis younghusbandi*. (**A**) Shannon index of water microbiota. (**B**) Shannon index of fish gut microbiota. Difference analysis was performed using one-way analysis of variance (ANOVA) to assess the significance of alpha diversity indices across different seasons and regions. Tukey’s HSD post hoc tests were applied to identify specific group differences. Significant differences are marked with “a, b, c, d”; no common superscript denotes a significant difference (*p* < 0.05). (**C**) Principal Coordinates Analysis (PCoA) of water microbial communities. (**D**) PCoA of fish gut microbial communities. The statistical significance of the dispersions between groups is calculated by PERMANOVA (R^2^ and P). Triple asterisks (***) denote an extremely significant difference (*p* < 0.001).

As shown in Figure 1, microbial α-diversity in both culture waters and fish guts varied significantly among treatments. In culture waters (Figure 1A), the Shannon indices of bacteria, protozoa, algae, and fungi were all significantly affected by culture modes. Bacterial diversity peaked in the WLW group, whereas protozoan, algal, and fungal diversities were consistently highest in the PW group (*p* < 0.05), indicating strong habitat-specific differentiation among microbial domains. In the fish gut (Figure 1B), microbial diversity patterns also differed markedly among treatments. Bacterial diversity was highest in the WLF and RF groups, protozoan diversity was lowest in the RCF group, and algal and fungal diversities peaked in the WLF group, demonstrating treatment-dependent restructuring of gut microbial diversity across multiple taxonomic groups. PCoA based on Bray–Curtis distances further revealed clear treatment-driven shifts in community structure (Figure 1C,D). In culture waters, bacterial communities showed the strongest separation among treatments, indicating high sensitivity to environmental variation, while protozoan communities also displayed distinct treatment-specific clustering. Algal and fungal communities exhibited comparatively weaker but still discernible separation patterns. In the gut microbiota, bacterial and protozoan communities formed well-defined treatment clusters, whereas algal and fungal communities showed moderate but consistent treatment-associated differentiation. Together, these results demonstrate that different culture modes significantly shape both microbial diversity and community structure in culture waters and fish guts, with bacteria and protozoa exhibiting the highest responsiveness to environmental and management-driven changes.

### 3.3. Composition and Differences in Microbial Communities in Culture Waters and Fish Guts

**Figure 2:**
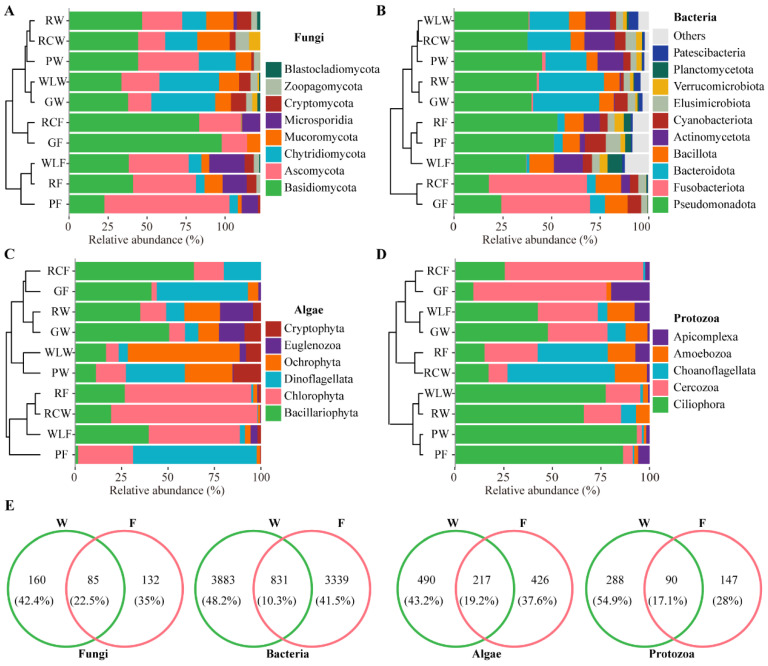
Microbial species composition in aquaculture water environments and the gut of *Schizopygopsis younghusbandi.* (**A**) Relative abundance of fungal phyla in water and gut microbiota. (**B**) Relative abundance of bacterial phyla in water and gut microbiota. (**C**) Relative abundance of algal phyla in water and gut microbiota. (**D**) Relative abundance of protozoan phyla in water and gut microbiota. (**E**) Venn diagram analysis of microbial species in aquaculture water and fish guts.

Figure 2 summarizes the taxonomic composition and relative abundance of microbial communities under different treatments (Tables S1–S4). At the phylum level, fungal communities were consistently dominated by Ascomycota and Basidiomycota across samples, although their relative proportions varied markedly among treatments, indicating strong environmental and host-associated effects on fungal community assembly (Figure 2A). Bacterial communities were primarily composed of Pseudomonadota, Bacteroidota, and Bacillota (Figure 2B), with Pseudomonadota representing a core and dominant component in most samples, while Bacteroidota and Bacillota showed treatment-dependent enrichment patterns. Other bacterial phyla generally occurred at low relative abundance and were sporadically enriched in specific samples. Algal communities were dominated by Chlorophyta and Bacillariophyta (Figure 2C), with reciprocal dominance patterns across treatments, suggesting differential regulation by environmental conditions such as nutrient availability and light regimes. Protozoan communities were mainly composed of Ciliophora and Cercozoa (Figure 2D), whose relative abundances varied substantially among treatments, reflecting treatment-specific restructuring of the micro-food web. Venn analysis revealed pronounced compositional differentiation between water (W) and fish gut (F) communities across all microbial groups (Figure 2E). Although a subset of taxa was shared, each habitat was primarily characterized by a large proportion of unique species, particularly in bacteria, where shared taxa accounted for only 10.3% of total species, indicating strong habitat filtering and niche differentiation between aquatic and gut environments.

### 3.4. Associations Between Microbial Community Composition and Environmental Factors

**Figure 3:**
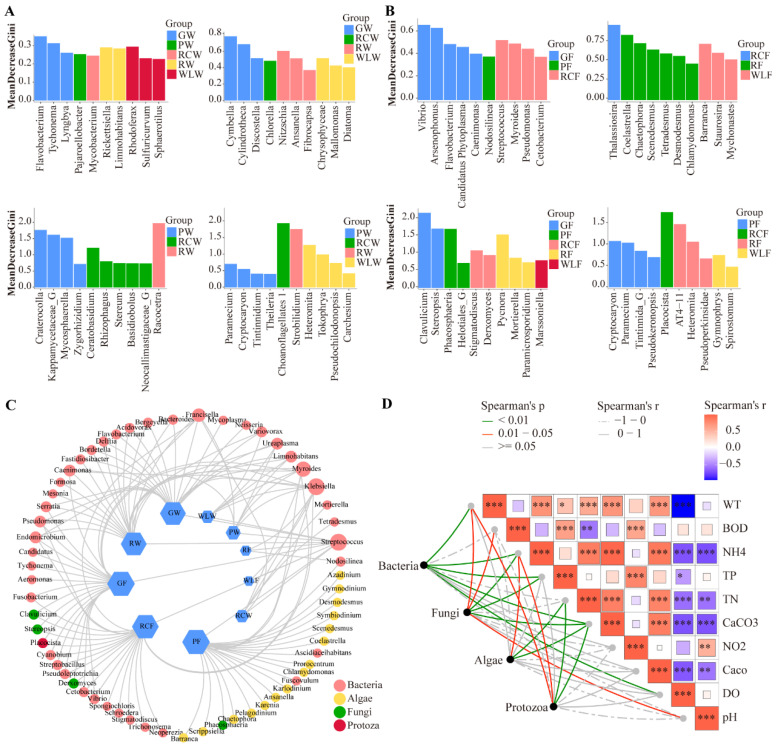
Microbial community structure in aquaculture water environments and fish guts, and their relationships with environmental factors. (**A**) Key genus-level microbial taxa distinguishing aquaculture modes identified by Random Forest analysis. Feature importance was calculated using the Mean Decrease in Gini index. (**B**) Key genus-level microbial taxa distinguishing aquaculture modes in fish gut microbiota identified by Random Forest analysis, with feature importance evaluated using the Mean Decrease in Gini index. (**C**) Co-occurrence network analysis of microbial communities in aquaculture water and fish guts. (**D**) Mantel analysis between microbial communities and environmental factors in aquaculture systems. A single asterisk (*) denotes a significant difference (*p* < 0.05), double asterisks (**) denote a highly significant difference (*p* < 0.01), and triple asterisks (***) denote an extremely significant difference (*p* < 0.001).

**Figure 4:**
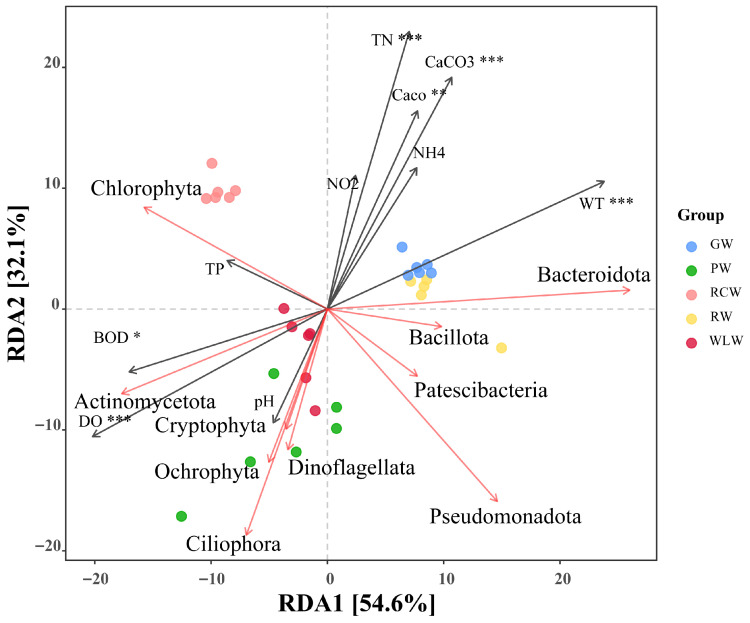
RDA analysis of environmental factors in water and fish gut microbiota under different aquaculture modes. A single asterisk (*) denotes a significant difference (*p* < 0.05), double asterisks (**) denote a highly significant difference (*p* < 0.01), and triple asterisks (***) denote an extremely significant difference (*p* < 0.001).

Random forest analysis revealed distinct microbial indicator taxa discriminating among culture water treatments (Figure 3A). Key bacterial taxa exhibited strong environmental specificity, with Flavobacterium enriched in the GW group, Sphingomonas dominating the WLW group, and Limnohabitans preferentially associated with the PW group. Algal and diatom taxa also showed treatment-specific patterns, including enrichment of Cymbella in GW, Chlorella in PW, and Cryptomonas in RCW, while Craticula and Gyrosigma were primarily associated with the PW group. Protozoan indicators similarly displayed distinct distributions, with Paramecium enriched in PW and Amoeba showing higher abundance in WLW and RCW, indicating strong environmental filtering effects on community assembly. In fish gut communities, random forest analysis identified treatment-specific indicator taxa (Figure 3B). Vibrio was predominantly enriched in the GF group, while Flavobacterium and Pseudomonas were associated with PF and RCF. Sphingomonas and Bacillus were characteristic of the WLF group, accompanied by distinct algal, diatom, fungal, and protistan assemblages showing clear treatment-dependent differentiation, collectively indicating that culture mode reshaped gut microbial composition through host–environment interactions. Co-occurrence network analysis further demonstrated treatment-dependent restructuring of microbial interaction patterns (Figure 3C). Bacterial taxa occupied central hub positions and formed complex synergistic and antagonistic interactions with algae, fungi, and protozoa. The GW–GF and RCW–RCF treatments exhibited higher network density and connectivity, indicating stronger microbial interactions, functional complementarity, and community stability, whereas the WLW–WLF treatment showed reduced connectivity, suggesting weakened network robustness and ecological stability. Spearman correlation analysis revealed significant associations between major microbial taxa and environmental variables (Figure 3D). Bacterial communities were positively correlated with TP, TN, and DO, algae were positively associated with pH and DO, fungi were negatively correlated with NO_2_^−^ and CaCO_3_, and protozoa showed negative correlations with BOD_5_ and NH_4_^+^ (*p* < 0.05). These results indicate that nutrient availability, oxygen conditions, and organic load are key drivers shaping cross-domain microbial community structure and interaction networks across culture systems. The RDA results further indicated that the first ordination axis explained 54.6% of the variation, while the second axis accounted for 32.1% (Figure 4). Together, these two axes explained 86.7% of the variation in gut microbial community composition. Total nitrogen (TN), CaCO_3_, CaO, and water temperature (WT) were identified as significant explanatory variables and were the primary factors driving changes in community structure.

### 3.5. Co-Occurrence Network Structure and Topological Characteristics of Microbial Communities in Culture Waters and Fish Guts

**Figure 5:**
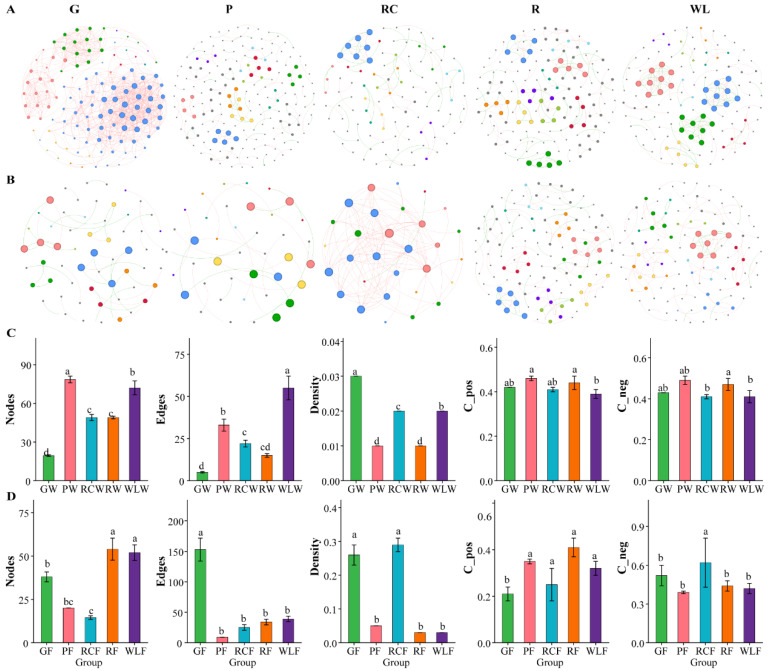
Co-occurrence network structure and topological feature analysis of microbial communities in different aquaculture water environments and fish guts. (**A**) Co-occurrence network structure analysis of water microbiota. (**B**) Co-occurrence network structure analysis of fish gut microbiota. (**C**) Statistical analysis of topological parameters of the co-occurrence network of water microbiota. (**D**) Statistical analysis of topological parameters of the co-occurrence network of fish gut microbiota. Significant differences are marked with “a, b, c, d”; no common superscript denotes a significant difference (*p* < 0.05).

Co-occurrence network analysis revealed pronounced treatment-dependent differences in microbial interaction structures in both water and gut environments (Figure 5). In water communities, the GW and WLW treatments formed the most complex and highly connected networks, characterized by increased node number, edge number, and network density, indicating intensified microbial interactions and stronger community integration. In contrast, the PW and RCW treatments exhibited simplified network structures with reduced connectivity, while the RW treatment showed an intermediate interaction pattern. In gut communities, the RCF and RF treatments displayed the highest network complexity, with significantly increased connectivity and interaction density, reflecting enhanced microbial co-occurrence and community integration. By contrast, the GF and PF treatments formed simplified networks with reduced interaction strength, whereas the WLF treatment exhibited moderate network complexity. Overall, gut microbial networks were structurally less complex than water networks, consistent with host filtering effects and ecological niche restriction within the intestinal environment. Topological parameter analysis further confirmed these patterns. In water networks, the GW group showed significantly higher node number and network density than all other treatments (*p* < 0.05), indicating the most tightly connected microbial structure, followed by the WLW group. The PW, RCW, and RW groups exhibited significantly lower network complexity. In gut networks, the RCF and RF groups had significantly higher node number, edge number, and network density than other treatments (*p* < 0.05), whereas the GF and PF groups showed the lowest values, with WLF at intermediate levels. The proportions of positive and negative correlations were broadly comparable among treatments, with only minor variation, indicating that network restructuring was primarily driven by changes in interaction strength and connectivity rather than interaction direction.

## 4. Discussion

### 4.1. Effects of Aquaculture Water Environments on the Growth and Survival of Schizopygopsis younghusbandi

Fish growth and survival are jointly regulated by genetic background and environmental conditions, with water temperature, dissolved oxygen, and nutrient availability representing key ecological drivers in aquaculture systems [39,40]. Artificial regulation of these factors has been widely demonstrated to improve growth efficiency and health status in cultured fish. Previous studies have reported strong species-specific responses to environmental conditions, including salinity and temperature regulation in Anguilla marmorata [41] and superior performance of *Triplophysa yarkandensis* in saline–alkaline systems [42]. Consistent with these findings, the present study demonstrates that aquaculture water environments significantly influence the growth performance of *S. younghusbandi*. The RCF group exhibited the highest growth rate, whereas the WLF group showed the lowest, with significant inter-treatment differences. This pattern is primarily attributable to the ectothermic physiology of fish: the recirculating aquaculture system provided higher and more stable temperatures, reducing thermal fluctuations and enhancing feeding activity and feed conversion efficiency, thereby promoting rapid growth. In contrast, the wetland system (WLF) exhibited the lowest survival rate, likely due to prolonged exposure to low-temperature conditions, which can impair immune function, increase disease susceptibility, and elevate stress-induced mortality risk. This interpretation is consistent with the findings of He. [43], who reported that sustained low temperatures significantly increase physiological stress in Schizothorax species despite broad thermal tolerance ranges. Collectively, these results indicate that temperature stability and moderate thermal regulation are critical prerequisites for sustainable aquaculture of *S. younghusbandi* in plateau environments. A limitation of this study is that the feed conversion ratio (FCR) was not recorded, preventing a direct evaluation of feed utilization efficiency. Although growth performance and microbiome functional profiles may provide indirect insights, future studies should include feed intake measurements to enable a more comprehensive assessment.

### 4.2. Effects of Aquaculture Water Bodies on Intestinal Microbial Diversity of Schizopygopsis younghusbandi

Across Aquatic environments and fish intestines host complex microbial communities that play essential roles in host metabolism, immunity, and disease resistance [23].

Its functions primarily include fermenting dietary components, producing a variety of metabolites, regulating metabolic pathways, and influencing the host’s energy acquisition efficiency [44]. In this study, significant differences in bacterial, protozoan, algal, and fungal diversity were observed among aquaculture systems, indicating strong environmental structuring effects on microbial communities. This may be an important factor contributing to growth differences under different aquaculture systems. Nie Z et al. also demonstrated in common carp that there were significant differences in gut microbial composition between fast-growing and slow-growing individuals [45]. Although bacterial diversity was highest in the WLW group, this was accompanied by elevated mortality, suggesting that high microbial diversity alone does not guarantee ecosystem stability or host health, and that functional composition and environmental stress levels are equally critical determinants. Higher protozoan, algal, and fungal diversity in the PW system suggests that physicochemical conditions and management practices increased resource heterogeneity and micro-niche availability, consistent with patterns reported in Qinghai-Xizang wetland ecosystems [46]. PCoA analysis further demonstrated clear community differentiation among treatments, with bacterial communities showing the strongest sensitivity to environmental variation, followed by protozoa, while algae and fungi exhibited relatively weaker but still detectable separation, suggesting that microbial responses to environmental change are closely linked to their ecological roles and niche adaptability. At the intestinal level, aquaculture environments significantly affected both α- and β-diversity of gut microbial communities, indicating that habitat variation not only influenced within-sample diversity but also drove pronounced between-group community dissimilarity, which is consistent with previous findings in aquaculture fish species showing that environmental heterogeneity is a key driver of gut microbiota variation across rearing systems. Notably, despite these significant shifts in β-diversity, a stable core microbiota was still observed across treatments, suggesting that certain microbial taxa are consistently maintained in the fish intestine regardless of environmental changes, likely due to their essential roles in host physiological processes such as nutrient metabolism, immune regulation, and intestinal homeostasis; similar patterns of core microbiota stability have also been reported in other important aquaculture fish species, highlighting the evolutionary and functional conservation of host-associated microbial symbionts. In addition, variations in gut microbial composition and diversity were closely associated with differences in growth performance among treatments, supporting the idea that gut microbiota may contribute to host growth regulation through effects on nutrient utilization and metabolic efficiency, which is also consistent with previous studies reporting links between microbial community shifts and growth variation in cultured fish [47]. Intestinal bacteria showed the strongest treatment separation, highlighting the interaction between environmental microbial inputs, husbandry practices, and host filtering mechanisms in shaping gut microbial steady states. Protozoa exhibited stable clustering but reduced diversity under some treatments, suggesting their potential role as sensitive indicator taxa influencing bacterial stability via trophic interactions. Algae and fungi displayed weaker but treatment-specific responses, likely reflecting ingestion-mediated transport and transient colonization regulated by gut microenvironmental conditions. These patterns are consistent with previous observations in Coilia species [48]. Overall, aquaculture water environments and management regimes drive microbial community restructuring at both α- and β-diversity levels, with bacteria and protozoa showing the highest sensitivity, while algae and fungi respond more indirectly and over longer temporal scales. This study found that changes in the aquaculture environment significantly altered the overall composition of the gut microbiota; however, the core microbiota remained largely stable. These core microbial taxa are typically composed of relatively abundant genera and may form close mutualistic relationships with the host. They are involved in fundamental physiological processes such as nutrient metabolism, immune regulation, and maintenance of the intestinal barrier, and are therefore less susceptible to environmental fluctuations.

### 4.3. Effects on Gut Microbial Community Composition and Co-Occurrence Networks

Consistent with previous studies [49,50], the present results indicate that aquaculture environments and host selection jointly regulate microbial community assembly in both water and gut ecosystems. Dominance patterns of major microbial phyla reflect functional structuring driven by organic matter availability, nutrient cycling, and trophic interactions. Low species overlap between water and gut communities, particularly for bacteria, indicates strong niche filtering and differentiated microbial source inputs. Machine learning analyses further identified treatment-specific indicator taxa, reflecting coordinated shifts in bacterial decomposition, algal primary production, and protozoan grazing pressure, leading to distinct microbial food web configurations. Co-occurrence network analysis revealed that bacteria occupied central hub positions and formed complex interaction networks with algae, fungi, and protozoa. Denser and more interconnected networks in GW–GF and RCW–RCF treatments suggest enhanced functional complementarity and potential system stability, whereas reduced connectivity in WLW–WLF networks indicates weakened interaction chains and lower resilience. Environmental correlations further identified nutrients (TP, TN), dissolved oxygen, and organic loading as key drivers shaping cross-domain microbial interactions. Network topology analysis showed that differences among treatments were primarily driven by changes in interaction strength and connectivity rather than interaction direction, indicating that management practices restructure microbial systems mainly by regulating network integration. Water networks exhibited higher complexity than gut networks, reflecting strong host filtering and niche constraints in the intestinal environment. Treatments promoting higher network connectivity and moderate positive interactions were associated with enhanced microbial coexistence and functional complementarity, whereas simplified networks reflected reduced interaction stability. Although co-occurrence networks represent statistical associations rather than direct causality [51], these results highlight the importance of maintaining high connectivity and balanced interactions to promote stability and resilience in both aquatic and gut microbiomes.

## 5. Conclusions

*Schizopygopsis younghusbandi* is an important indigenous economic fish species in Xizang and is highly sensitive to environmental conditions under artificial culture. This study demonstrates that different aquaculture systems significantly affect fish growth performance and are closely associated with alterations in both intestinal and environmental microbial communities, indicating a strong linkage between habitat conditions, microbiota composition, and host physiological responses. In particular, the observed associations between growth-related parameters and gut microbiota variation suggest that microbial communities may play an important role in regulating nutrient metabolism and energy utilization, thereby contributing to differences in growth performance among aquaculture systems. Notably, although significant changes in β-diversity were observed across environments, a stable core microbiota was consistently detected in the intestine, suggesting that certain microbial taxa are resilient to habitat variation and may be essential for maintaining fundamental metabolic functions and host homeostasis. Recirculating aquaculture systems (RAS), in particular, appear to provide more stable environmental conditions that may support a more balanced microbial structure and promote better fish growth performance compared to other culture modes. Therefore, future aquaculture development should prioritize RAS or ecologically optimized systems with environmental regulation capacity, while management strategies should focus on maintaining microbial homeostasis through coordinated control of nutrients, dissolved oxygen, and organic loading. Overall, these findings provide important insights into the interactions among the aquaculture environment, gut microbiota, and growth performance, offering a scientific basis for the conservation, sustainable utilization, and industrial development of *Schizopygopsis younghusbandi* in the Qinghai–Xizang Plateau.

## Figures and Tables

**Table 1:** Physicochemical parameters of water quality and analytical methods.

Physicochemical Parameters	Analytical Method	Method Detection Limit
Water temperature (WT, °C)	YSI multiparameter water quality analyzer (GB/T 13195-1991)	/
Dissolved oxygen (DO, mg L^−1^)	YSI multiparameter water quality analyzer (HJ 505-2009)	/
pH	YSI multiparameter water quality analyzer (HJ 1147-2020)	/
Biochemical oxygen demand (BOD_5_, mg L^−1^)	Dilution and seeding method (HJ 535-2009)	0.5 mg L^−1^
Total nitrogen (TN, mg L^−1^)	Alkaline potassium persulfate digestion–UV spectrophotometry (HJ 636-2012)	0.05 mg L^−1^
Total phosphorus (TP, mg L^−1^)	Ammonium molybdate spectrophotometry (GB 11893-89)	0.01 mg L^−1^
Ammonia nitrogen (NH_4_^+^-N, mg L^−1^)	Sodium salicylate spectrophotometry (HJ 535-2009)	0.025 mg L^−1^
Nitrite nitrogen (NO_2_^−^-N, mg L^−1^)	Spectrophotometric method (GB 7493-87)	0.001 mg L^−1^
Total hardness (as CaCO_3_, mg L^−1^)	EDTA titration (GB 7477-87)	5 mg L^−1^
Total alkalinity (as CaCO_3_, mg L^−1^)	Acid titration method (GB 7477-87)	/

**Table 2:** Physicochemical Indicators of Different Aquaculture Water Environments.

Water Quality Parameters	PW	WLW	GW	RW	RCW
WT	6.91 ± 0.02 ^b^	5.33 ± 0.02 ^a^	11.72 ± 0.01 ^d^	8.74 ± 0.04 ^c^	13.89 ± 0.02 ^e^
BOD	3.70 ± 0.03 ^e^	1.93 ± 0.02 ^b^	0.70 ± 0.02 ^a^	3.39 ± 0.02 ^d^	3.23 ± 0.02 ^c^
NH4	0.29 ± 0.01 ^b^	0.08 ± 0.00 ^a^	0.37 ± 0.00 ^d^	0.36 ± 0.00 ^d^	0.35 ± 0.00 ^c^

Note: Different letters indicate significance levels (ANOVA Duncan multiple comparisons, ≤0.05 was considered statistically significant).

**Table 3:** Effects of Different Aquaculture Water Environment Systems on the Growth of *Schizopygopsis younghusbandi*.

Fish Growth Performance Index	PF	WLF	GF	RF	RCF
Initial body weight/g	49.77 ± 2.03	50.59 ± 1.62	51.67 ± 1.56	50.13 ± 1.97	50.92 ± 2.26
Final body weight/g	77.76 ± 2.29 ^a^	86.75 ± 13.72 ^ab^	94.94 ± 14.32 ^b^	91.84 ± 19.91 ^b^	115.39 ± 17.19 ^c^
Initial body length/cm	15.64 ± 0.72	15.49 ± 1.10	15.48 ± 1.08	15.22 ± 1.38	15.45 ± 1.07
Final body length/cm	19.04 ± 0.97 ^ab^	18.06 ± 1.12 ^a^	19.62 ± 1.54 ^b^	19.28 ± 1.40 ^ab^	20.09 ± 1.60 ^b^
WGR/%	56.32 ± 2.29 ^a^	70.87 ± 21.85 ^ab^	83.17 ± 22.28 ^b^	82.07 ± 32.77 ^b^	125.74 ± 24.75 ^c^
SGR/(%/d)	0.37 ± 0.02 ^a^	0.44 ± 0.07 ^ab^	0.50 ± 0.10 ^b^	0.49 ± 0.15 ^b^	0.67 ± 0.05 ^c^
CF/(g/cm^3^)	1.14 ± 0.14 ^a^	1.46 ± 0.07 ^c^	1.26 ± 0.14 ^b^	1.26 ± 0.06 ^b^	1.43 ± 0.13 ^c^
Survival rate/%	90.67 ± 0.58 ^a^	91.33 ± 1.53 ^a^	95.33 ± 1.53 ^b^	95.67 ± 1.53 ^b^	95.67 ± 2.08 ^b^

Note: Different letters indicate significance levels (ANOVA Duncan multiple comparisons, significance level 0.05).

## Data Availability

The original contributions presented in the study are included in the article/Supplementary Materials. Further inquiries can be directed to the corresponding author.

## Supplementary Materials

The following supporting information can be downloaded at: https://www.mdpi.com/article/10.3390/microorganisms14040925/s1, Table S1: Abundance of Protozoa in water and fish gut under different aquaculture modes; Table S2: Abundance of Bacteria in water and fish gut under different aquaculture modes; Table S3: Abundance of Algae in water and fish gut under different aquaculture modes; Table S4: Abundance of Fungi in water and fish gut under different aquaculture modes.
